# Evaluating the Potential Value of Natural Product Cuminic Acid against Plant Pathogenic Fungi in Cucumber

**DOI:** 10.3390/molecules22111914

**Published:** 2017-11-06

**Authors:** Yong Wang, Jie Zhang, Yang Sun, Juntao Feng, Xing Zhang

**Affiliations:** Research and Development Center of Biorational Pesticides, Northwest A & F University, Yangling 712100, China; wy2010102163@163.com (Y.W.); deit1984@sina.com (J.Z.); Sunyang136592@nwsuaf.edu.cn (Y.S.); jtfeng@126.com (J.F.)

**Keywords:** botanical fungicide, cuminic acid, cucumber, *F. oxysporum*, *C. lagenarium*, control efficacy

## Abstract

Fusarium wilt and anthracnose are two major diseases which limit the yield and quality of cucumber worldwide. Cuminic acid was extracted from the seed of *Cuminum cyminum* L. The mean EC_50_ values of cuminic acid for inhibiting mycelial growth and zoospore germination of five *Fusarium oxysporum* f. sp. *cucumerinum* strains were 25.66 ± 3.02 μg/mL and 15.99 ± 2.19 μg/mL, and of four *Colletotrichum lagenarium* (Pass.) Ellis and Halsted strains were 29.53 ± 3.18 μg/mL and 18.41 ± 2.78 μg/mL, respectively. In greenhouse experiments, cuminic acid at 2000 μg/mL exhibited 70.77% protective and 62.63% curative efficacies against *F. oxysporum*, and 65.43% protective and 55.46% curative efficacies against *C. lagenarium*. Moreover, the translocation behavior of cuminic acid, determined by high performance liquid chromatography (HPLC), showed that it could be readily absorbed and transported upward and downward in cucumber. Importantly, superoxide dismutase (SOD) and pyphenol oxidase (PPO) activities of cucumber leaves treated with cuminic acid increased significantly. All results indicated that cuminic acid showed antifungal activity, and could be used as a botanical fungicide in disease management. This study encourages further investigation on the mechanism of action of cuminic acid and the development of alternative antifungal drugs.

## 1. Introduction

Cucumber is one of the most important vegetables grown in the world, especially in China. Fusarium wilt, a soil-borne disease caused by *F. oxysporum*, and anthracnose, which is caused by *C. lagenarium*, are the main diseases that limit the yield and quality of cucumber worldwide [1,2]. Stems and roots, and especially root wounds, can be infected by *F. oxysporum.* The chlamydospores generated by *F. oxysporum* can survive in infested field soil for several years [3]. On the other hand, all above-ground tissues of cucumber, such as leaves, stems, petioles, and fruits, can be infected by *C. lagenarium* [4].

In practice, the application of chemical fungicides is still the main method used for disease management in cucumber due to the limit of highly resistant cultivars [5,6]. However, widespread use of these chemical fungicides has generated serious problems, including cost, environmental pollution, decreased efficacy due to pathogen resistance, and threats to human health [7,8]. Therefore, screening for alternative fungicides that do not harm people or the environment is urgently necessary. Considering the requirements of modern greenhouse agriculture, botanical fungicides have attracted more attention. Indeed, the practice of using botanical fungicides, or plant extracts as we know them in agriculture, dates back at least two millennia in many countries, such as ancient China, Greece, India, Egypt, and North America [9]. Botanical fungicides have certain advantages: They usually break down rapidly and do not persist in the environment, and have a low risk of inducing pathogen resistance. Moreover, botanical fungicides can also be used as lead compounds for the synthesis of novel compounds [10,11].

Cuminic acid was extracted from the seed of *Cuminum cyminum* L. in our previous research [12]. Cuminic acid belongs to the benzoic acid chemical group. Previous studies have shown that cuminic acid exhibits obvious antifungal activity against several plant pathogens, such as *Phytophthora capsici* Leonian, *Rhizoctonia cerealis* E.P. van der Hoeven, and *Sclerotinia sclerotiorum* (Lib.) de Bary. The mycelial growth of *S. sclerotiorum* and *R. cerealis* was completely inhibited when treated with cuminic acid at 200 μg/mL [13,14]. Moreover, in greenhouse experiments, more than 50% efficacy against *S. sclerotiorum* and *Blumeria graminis* f. sp. *tritici* was obtained when plants were treated with cuminic acid at 1000 μg/mL, which was comparable with the efficacy of procymidone at 100 μg/mL [13]. Moreover, the EC_50_ value of cuminic acid for inhibiting the mycelial growth of *P. capsici* was only 14.54 µg/mL, which was even lower than the EC_50_ value of the natural compound eugenol against *P. capsici* [12].

Cuminic acid has demonstrated broad-spectrum antifungal activity. However, few reports about the antifungal activity of cuminic acid against *F. oxysporum* or *C. lagenarium* are available in the literature. Therefore, the aim of this study was to evaluate the potential value of the natural compound cuminic acid, extracted from the seed of *Cuminum cyminum* L., as a botanical fungicide against *F. oxysporum* or *C. lagenarium*.

## 2. Results

### 2.1. Sensitivity to Cuminic Acid

There was significant difference (*p* = 0.05) in sensitivity to cuminic acid between strains (Table 1). The EC_50_ values for the inhibition of mycelial growth for the five *F. oxysporum* strains and four *C. lagenarium* strains by cuminic acid ranged from 18.46 to 32.34 µg/mL and 22.45 to 35.71 µg/mL, with mean EC_50_ values of 25.66 and 29.53 µg/mL, respectively. The EC_50_ values for the inhibition of spore germination of the five *F. oxysporum* strains and four *C. lagenarium* strains by cuminic acid ranged from 13.37 to 18.22 µg/mL and 17.44 to 20.45 µg/mL, with mean EC_50_ values of 15.99 and 18.41 µg/mL, respectively. However, the EC_50_ values for cuminic acid were always higher than that of carbendazim, for both inhibiting mycelial growth and spore germination (Table 1).

### 2.2. Protective and Curative Activity of Cuminic Acid

**Figure 1:**
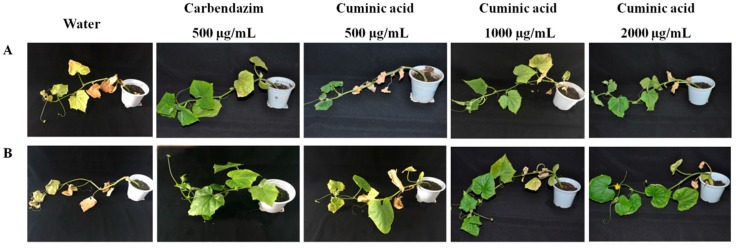
(**A**) Protective and (**B**) curative activity of cuminic acid against *F. oxysporum*.

**Figure 2:**
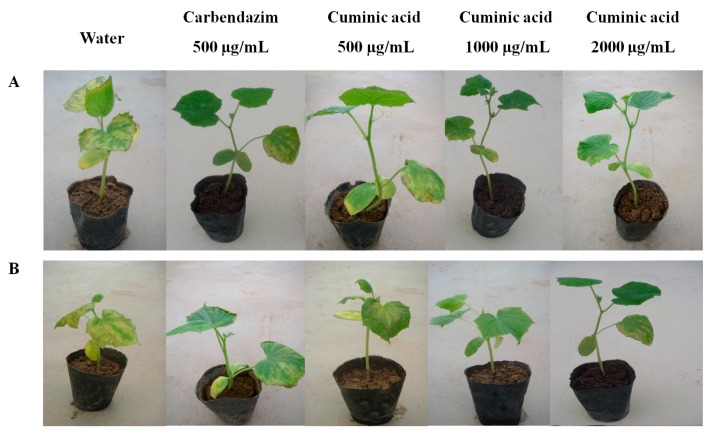
(**A**) Protective and (**B**) curative activity of cuminic acid against *C. lagenarium*.

Protective and curative activities of cuminic acid against *F. oxysporum* and *C. lagenarium* were determined in a greenhouse. The results showed that cuminic acid exhibited both protective and curative activities against both *F. oxysporum* and *C. lagenarium.* When treated with cuminic acid at 2000 µg/mL, the protective efficacies against *F. oxysporum* and *C. lagenarium* were 70.77% and 65.43%, respectively, which were even better or nearly equal to the efficacy attained by carbendazim at 500 µg/mL (Table 2 and Table 3). Moreover, the protective and curative efficacies of cuminic acid at 1000 and 2000 µg/mL against *F. oxysporum* were always higher than those against *C. lagenarium* treated with cuminic acid at the same concentration. The protective efficacies were always higher than curative efficacies whether treated with cuminic acid at 500, 1000 or 2000 µg/mL (Figure 1 and Figure 2). This indicates that cuminic acid not only has a better protective than curative activity, but also has better antifungal activity against *F. oxysporum* than *C. lagenarium.*

### 2.3. Translocation Behavior of Cuminic Acid

**Figure 3:**
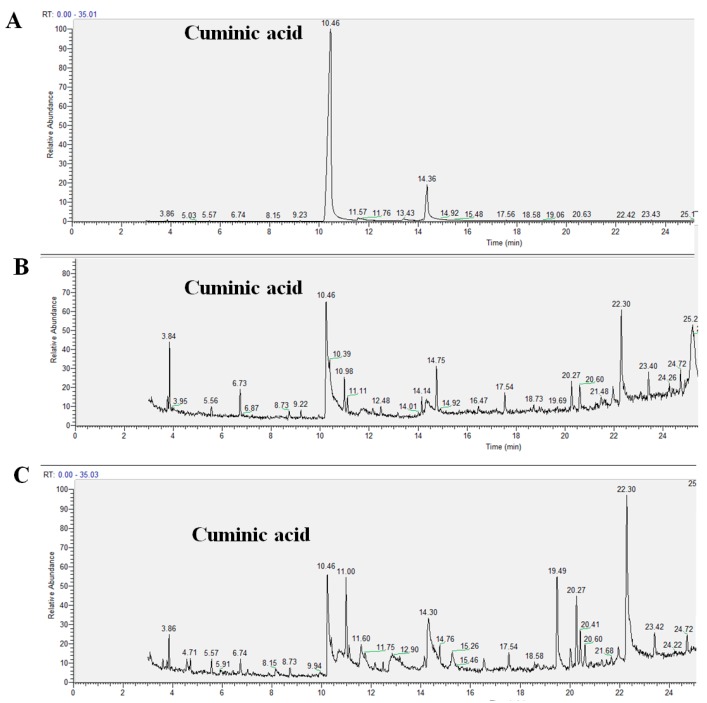
Detection of cuminic acid by HPLC. (**A**) Standard sample of cuminic acid; (**B**) cuminic acid in cucumber leaves from soil by irrigation; and (**C**) cuminic acid in cucumber roots from leaves by spraying.

As shown in Figure 3, the standard product of cuminic acid was detected after 10 min using liquid chromatography. As expected, cuminic acid was detected in both the roots of the cucumber plants sprayed with cuminic acid and the leaves of the cucumber plants irrigated with cuminic acid at the same time. LC-MS data for cuminic acid is shown in the supplementary materials. These results suggest that cuminic acid could be absorbed and translocated both upward and downward in cucumber plants.

### 2.4. *SOD*, *POD*, *PPO*, and *CAT* Activity

**Figure 4:**
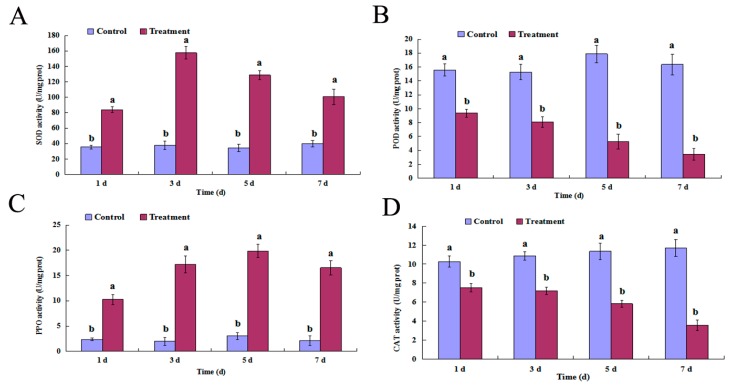
Detection of (**A**) superoxide dismutase (SOD); (**B**) peroxidase (POD); (**C**) polyphenol oxidase (PPO); (**D**) and catalase (CAT) activities of cucumber leaves treated with cuminic acid at 500 µg/mL. Values are means and standard errors; a, b mean values followed by different letter within the same day were significantly different in LSD tests at *p* = 0.05.

When treated with cuminic acid, the changes in enzyme activity were different. SOD and PPO activities increased over time and were always higher than the untreated control. SOD activity reached a maximum at three days, while PPO activity reached a maximum at five days. However, POD and CAT activities decreased over time and were always lower than the control (Figure 4).

## 3. Discussion

Plant diseases Fusarium wilt and anthracnose, caused by pathogenic fungi, are the most important factors that limit cucumber production worldwide, especially in China. In practice, most efforts to control the two diseases have focused on the application of synthetic chemical fungicides [5,6]. However, serious problems, such as handling hazards, fungicide resistance, and threats to human health and the environment, have generated concern. Therefore, looking to botanical fungicides or plant extracts has started in an attempt to find alternatives. 

In our previous study, 1.2 g cuminic acid was extracted from 15 kg of *Cuminum cyminum* L. seed [15]. Importantly, the toxicity test of 1% cuminic acid micro-emulsion on animals and plants, such as quail, bee, silkworm, carp, earthworm, tadpole, wheat, chili, and oilseed rape, was performed. The results were as follows: the LD_50_ for quail was greater than 1000 mg/kg; for bees it was greater than 5000 mg/L; for silkworm it was greater than 1500 mg/L; for carp it was greater than 1600 mg/L; for earthworm it was greater than 1000 mg/L; for tadpoles it was greater than 40 mg/L; and it exhibited low risk for wheat, chili, and oilseed rape [16]. All the results suggested that cuminic acid was safe and would not biologically target any sensitive species.

In the current study, the antifungal activity of cuminic acid, which was extracted from the seed of *Cuminum cyminum* L., was assessed. The results showed that cuminic acid has obvious inhibitory activity against *F. oxysporum* and *C. lagenarium*. Moreover, cuminic acid was more active against spore germination than mycelial growth, which was consistent with its activity against *P. capsici* [12]. However, there was no significant difference among the EC_50_ values for carbendazim against mycelial growth or spore germination, indicating that the mechanism of action was different between cuminic acid and carbendazim. 

In a pathogen’s lifecycle, spore germination and mycelial growth are two different stages. Once infection has occurred, the suppression of mycelial growth within the host tissue becomes a major disease management target, which could interrupt the lifecycle of the pathogen [17]. In greenhouse experiments, cuminic acid at 2000 µg/mL exhibited over 60% protective and curative activity against *F. oxysporum*, which was nearly equal to the efficacy obtained by the chemical fungicide carbendazim, indicating that cuminic acid has both protective and curative activity. Therefore, it not only reduced the infection ability of the mycelia but also inhibited the spore germination. Cuminic acid was also detected in both the roots and leaves using HPLC, indicating that cuminic acid exhibits good characteristics of absorption and can be transported both upward and downward in cucumber. Cuminic acid could be used for the control of both foliar disease and root disease, which is different compared to the previous study that showed that carabrone could be only transported upward instead of downward [18].

Previous studies have demonstrated that plants have special immune sensors that can identify bacteria, viruses, fungi, and other microbial invasion. The enzyme system in plants can not only regulate the normal physiological activities, but also prevent the invasion of microorganisms [19,20]. In addition, salicylic acid and jasmonic acid are two important signal transmission compounds that induce plants’ defense systems [21,22]. In this study, the POD and CAT activity of the cucumber leaves decreased after being treated with cuminic acid, whereas the SOD and PPO activity increased significantly after being treated with cuminic acid. Work to explore the expression of genes correlated with the pathway of salicylic acid or jasmonic acid, which is associated with the plant’s defense ability, is underway in our lab. 

Integrated control of fungal diseases, including biological controls and safer chemicals such as food preservatives, chitosan, and plant-derived products, has been studied [23,24]. Benzoic acid has been commonly used as food preservative and is widespread in industrial wastewater [25]. Cuminic acid, extracted from the seed of *Cuminum cyminum* L., belongs to the benzoic acid chemical group. However, a previous study demonstrated that cuminic acid did not target organisms, was easily degradable in soil and water, and was environmentally friendly [26].

Over several decades, crop growers have generally apply synthetic fungicides as the main method for controlling plant diseases. However, modern organic agriculture, where products cultivated without applying any chemical pesticides, is increasing in popularity [27]. Natural plant chemicals, which are the major sources of industrial and medicinal materials, including plant extracts and essential oils, have also shown potential for agricultural pest management [28,29]. For example, *Vitex agnus-castus* extract exhibited strong antifungal activity against *Pythium ultimum* in tomatoes under both in vitro and in vivo conditions [30]; *Cortex Pseudolaricis* extract exhibited potential antifungal activity against *Colletotrichum gloeosporioides* [31]; and the essential oil from *Tetradium glabrifolium* fruits showed larvicidal activity against *Aedes albopictus* [32]. In addition, glucosinolates, terpenoids, flavor compounds, and glucosinolates are receiving increased attention worldwide [33]. Previous studies demonstrated that natural products could not only be directly used as crop protection agents, but could also be used as lead compounds for the synthesis of new pesticides, such as a series of strobilurin fungicides, including azoxystrobin and picoxystrobin, which were derived based on the structure of strobilurin A [34,35,36,37]. Considering the public concern about the impact of synthetic pesticides on human health and the environment, natural products extracted from microbes, plants, and other organisms will continue to be important sources for environmentally friendlier pest management tools.

## 4. Materials and Methods

### 4.1. Fungicides and Strains

Cuminic acid (98%) was purchased from Aladdin Bio-Chem Technology Company (Shanghai, China) and dissolved in 10 mL methanol to 100 mg/mL for stock solutions. Carbendazim (98%), provided by Shenyang Study Institute of Chemical Industry (Shenyang, China), was dissolved in 0.1 mol/L hydrochloric acid (HCl) at 10 mg/mL as stock solutions.

Five *F. oxysporum* strains and four *C. lagenarium* strains (single-spore isolates) were provided by the Research and Development Center of Biorational Pesticides, Northwest A & F University and maintained on potato dextrose agar (PDA) slants at 4 °C.

### 4.2. Sensitivity to Cuminic Acid

The EC_50_ values of cuminic acid for the inhibition of mycelial growth were determined according to a previous study [12]. Inverted mycelia plugs, 5 mm in diameter, were cut from the margin of the 5-day-old colonies and then transferred to PDA plates which were amended with cuminic acid at concentrations of 0, 6.25, 12.5, 25, 50 and 100 μg/mL. After the plates were incubated at 25 °C for 5 days, colony diameters in two perpendicular directions were measured and averaged. The inhibition rate of mycelial growth was calculated [12].

The activity of cuminic acid on the inhibition of spore germination was determined as per a prior study [37]. The strains were first incubated on PDA plates in the dark at 25 °C for 7 days. Then, the plates were drenched with 15 mL of sterile distilled water, and the spores were carefully collected from the culture surface. Spore suspensions for each strain, prepared at 1 × 10^5^, were spread on water agar plates amended with cuminic acid at 0, 3.125, 6.25, 12.5, 25 and 50 μg/mL. After incubation at 25 °C for 10 h, the germination of conidia was checked and the inhibition rate of spore germination was calculated. In terms of sensitivity, carbendazim was used as the control fungicide and the concentrations of carbendazim used were 0, 0.3125, 0.625, 1.25, 2.5, 5, 10, and 20 μg/mL. The experiment was conducted three times with three replicates per treatment. 

### 4.3. Protective and Curative Activity of Cuminic Acid in Greenhouse Experiments

The protective activity of cuminic acid against *F. oxysporum* or *C. lagenarium* was conducted as follows [30]. One cucumber plant per pot, at a similar growth stage (three to five leaves), were irrigated with 10 mL of water, and carbendazim at 500 μg/mL or cuminic acid at 500, 1000 or 2000 μg/mL. After 24 h, cucumber plants were irrigated with 10 mL of spore suspension (1 × 10^5^ spores) collected from *F. oxysporum*, or sprayed with 10 mL of spore suspension (1 × 10^5^ spores) collected from *C. lagenarium*.

Then, the irrigated plants were kept at 25 °C with 80% humidity in a growth chamber. All leaves from each plant and six plants for each concentration were used for the detection of anthracnose. The experiment was repeated three times. The disease indeices of Fusarium wilt and anthracnose were detected 15 days or 5 days after inoculation, respectively, and the control efficacy was calculated [8,38].

A 0–4 visual scale grade of the rhizomes and roots was used to determine the disease severity of *F. oxysporum*, where 0 represented rhizomes and roots with no symptoms, 1 denoted lesions on less than 25% of the total area, 2 denoted lesions on 25–50%, 3 denoted lesions on 50–75%, and 4 represented lesions on more than 75%. The disease index was calculated as 100 × [(n × 1 + n × 2 + n × 3 + n × 4)]/(4× total assessed plants), where n represents the number of diseased plants in that grade [38].

A 0–9 disease severity grade was used to test the disease severity of *C. lagenarium*, which was evaluated visually on individual leaves as the percentage of diseased area. 0 represented no symptoms; 1 indicated <1%, 3 indicated 1–10%, 5 indicated 10–25%, 7 indicated 25–50%, and 9 indicated >50%. The disease index was calculated as 100 × [(n × 1 + n × 3 + n × 5 + n × 7 + n × 9)]/(9 × total number of examined leaves), where n represents the numbers of leaves corresponding to the disease grade [8].

The equation to calculate control efficiency was:

Control efficacy (%) = [(disease index of control − disease index of treatment)/disease index of control] × 100

### 4.4. Translocation of Cuminic Acid in Cucumber Plants

To detect the downward translocation of cuminic acid, leaves of cucumber plants were sprayed with cuminic acid at 500 µg/mL until runoff. After the plants were incubated in a greenhouse for 3 days, rot of the plants were cut off and ground in liquid nitrogen. After being mixed with 5 mL of methanol, the solution was detected using high performance liquid chromatography (Waters Alliance 2690, Waters, Milford, MA, USA) [17]. For detecting the upward translocation of cuminic acid, cucumber plants were irrigated with 10 mL cuminic acid at 500 µg/mL. Then, the leaves were cut off and treated as described above. Cuminic acid (98%) was used as the standard product. The experiment was conducted twice with four plants per treatment.

HPLC detection conditions were as follows: Hpersil BDS-C_18_ (4.6 mm × 250 mm, 10 mm, Thermo Fisher Scientific, Waltham, MA, USA) was used. Seventy percent methanol was used as the mobile phase and the flow rate was 1 mL/min with an injection volume of 10 µL. The detection wavelength was 233 nm and the column was kept at room temperature.

### 4.5. Superoxide Dismutase, Peroxidase, Polyphenol Oxidase, and Catalase Activity 

All the leaves of cucumber plants at a similar growth stage were sprayed with water and cuminic acid at 500 µg/mL until runoff and then kept at 25 °C with 80% humidity. The plants were not inoculated with the pathogens. After incubation for 1, 3, 5 and 7 days, one leaf per plant was cut off (0.2 g per leaf) and the superoxide dismutase (SOD), peroxidase (POD), polyphenol oxidase (PPO), and catalase (CAT) activities were determined using commercial kits (Jiancheng, Nanjing, China) according to the manufacturer’s instructions. Six plants per treatment were used and the experiment was repeated three times.

### 4.6. Data Analysis

Due to the variances between experiments being homogeneous, data from repeated experiments were combined for analysis. Statistical analysis was conducted using SPSS 14.0 (SPSS Inc., Chicago, IL, USA). The EC_50_ values of the strains were calculated by linear regression of the log of the colony diameter versus fungicide concentration. The ANOVA procedure of SPSS and Fisher’s protected least significant difference (*p* = 0.05) were used to determine whether significant differences existed among the data.

## 5. Conclusions

This study demonstrated that the natural product cuminic acid not only demonstrated antifungal activity both against *F. oxysporum* and *C. lagenarium*, but also could be absorbed and transported upward and downward in cucumber. Importantly, cuminic acid altered the enzyme activities in cucumber leaves, which was consistent with our previous study that investigated the activity of cuminic acid against *P. capsici* [12]. Taken together, cuminic acid shows the potential to be used as a botanical fungicide in the management of plant diseases. Our efforts to induce cuminic acid-resistant mutants were unsuccessful, which indicated that these fungi might not readily develop resistance to cuminic acid. The work to explore the mechanism of action of cuminic acid and synthesize new compounds based on the chemical structure of cuminic acid requires further investigation.

## Figures and Tables

**Table 1:** Sensitivity to cuminic acid and carbendazim.

Strains ^a^	EC_50_ for Cuminic Acid (μg/mL)		EC_50_ for Carbendazim (μg/mL)	
	Inhibition of Growth	Inhibition of Germination	Inhibition of Growth	Inhibition of Germination
FO-1	21.37c ^b^	15.62b	0.900b	1.173c
FO-23	25.62c	13.37c	1.211b	1.432c
FO-12	18.46d	16.02ab	0.794b	0.983c
FO-14	32.34ab	16.73ab	0.869b	0.874c
FO-8	30.50b	18.22a	1.001b	0.992c
CL-32	22.45c	18.36a	7.322a	6.406a
CL-12	35.71a	19.43a	6.537a	6.035a
CL-7	30.09b	17.44a	5.459a	4.941b
CL-11	29.87b	20.45a	6.344a	6.075a

Note: ^a^ FO, *F. oxysporum*; CL, *C. lagenarium*; ^b^ a, b, c, d mean values followed by the different letter within the same column were significantly different in least significant difference (LSD) tests at *p* = 0.05.

**Table 2:** Protective and curative activity of cuminic acid against *F. oxysporum*.

Treatment	Protective Activity		Curative Activity	
	Disease Index	Efficacy (%)	Disease Index	Efficacy (%)
Cuminic Acid (500 µg/mL)	66.67b ^a^	26.16c	68.06b	23.43c
Cuminic Acid (1000 µg/mL)	51.39c	43.08b	50.00c	43.75b
Cuminic Acid (2000 µg/mL)	26.39d	70.77a	33.22d	62.63a
Carbendazim (500 µg/mL)	27.78d	69.22a	34.70d	60.96a
Water Control	90.28a	-	88.89a	-

Note: ^a^ a, b, c, d mean values followed by the different letter within the same column were significantly different in LSD tests at *p* = 0.05.

**Table 3:** Protective and curative activity of cuminic acid against *C. lagenarium*.

Treatment	Protective Activity		Curative Activity	
	Disease Index	Efficacy (%)	Disease Index	Efficacy (%)
Cuminic Acid (500 µg/mL)	42.80b ^a^	21.77c	36.01b	17.07c
Cuminic Acid (1000 µg/mL)	32.92c	39.85b	29.01c	33.18b
Cuminic Acid (2000 µg/mL)	18.93d	65.43a	19.34c	55.46a
Carbendazim (500 µg/mL)	18.31d	66.55a	16.05c	63.04a
Water Control	54.73a	-	43.42a	-

Note: ^a^ a, b, c, d mean values followed by the different letter within the same column were significantly different in LSD tests at *p* = 0.05.

## Supplementary Materials

Supplementary materials are available online.
