# Supplementary material for: Evaluating the Potential Value of Natural Product Cuminic Acid against Plant Pathogenic Fungi in Cucumber
Source: Molecules. 2017 Nov 6;22(11):1914. doi: 10.3390/molecules22111914 (PMC6150400; doi:10.3390/molecules22111914)
Supplement: Supplementary file 1 [file molecules-22-01914-s001.pdf]

molecules

Supplementary data: LC-MS Condition: High-resolution Ion Mobility LC-MS (AB SCIEX Triple TOF 5600+) Nexera X2, LC-30A (CTO-30A), 100% methanol was used as the mobile phase and the flow rate was 0.3 mL/min with an injection volume of 5 μL.

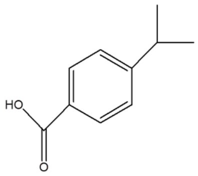

Figure S1. Chemical structure of cuminic acid.

Original data of LC-MS:

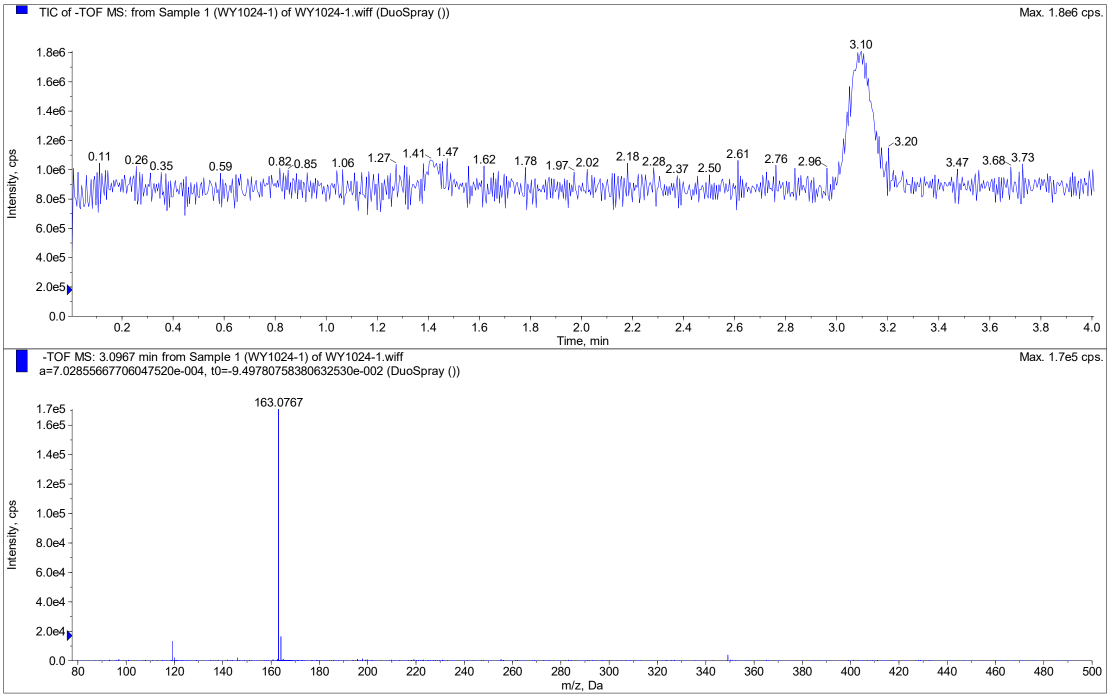

Figure S2. LC-MS data of standard compound cuminic acid (Total particle flow chart and MS figure).

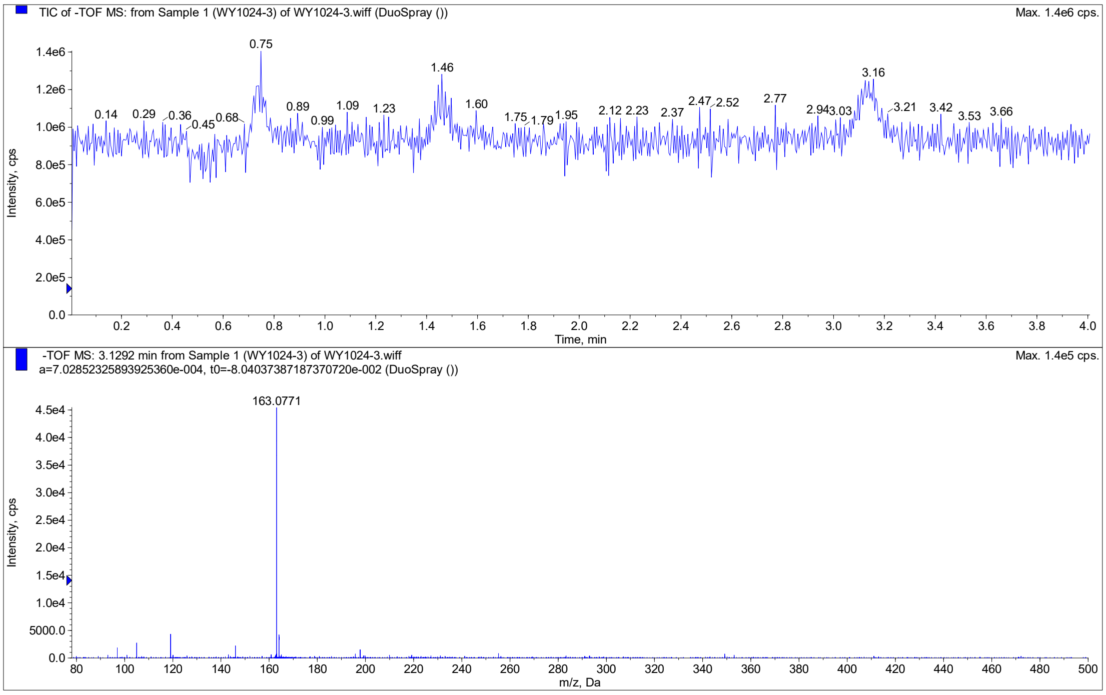

Figure S3. Cuminic acid in cucumber leaves from soil by irrigation

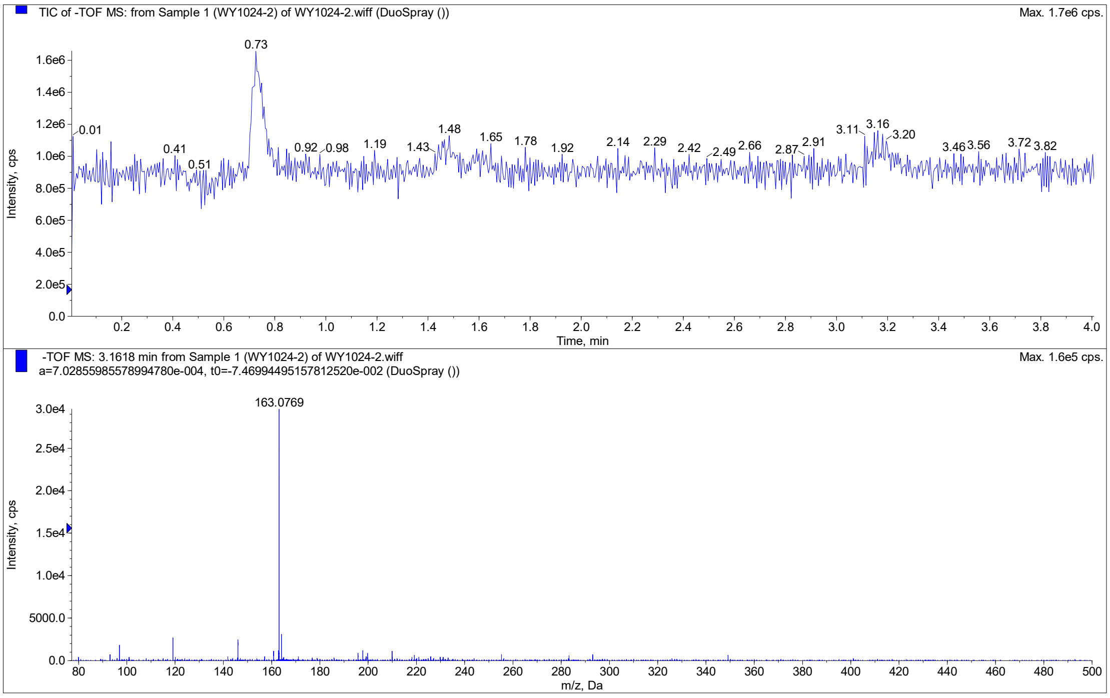

Figure S4. Cuminic acid in cucumber roots from leaves by spraying

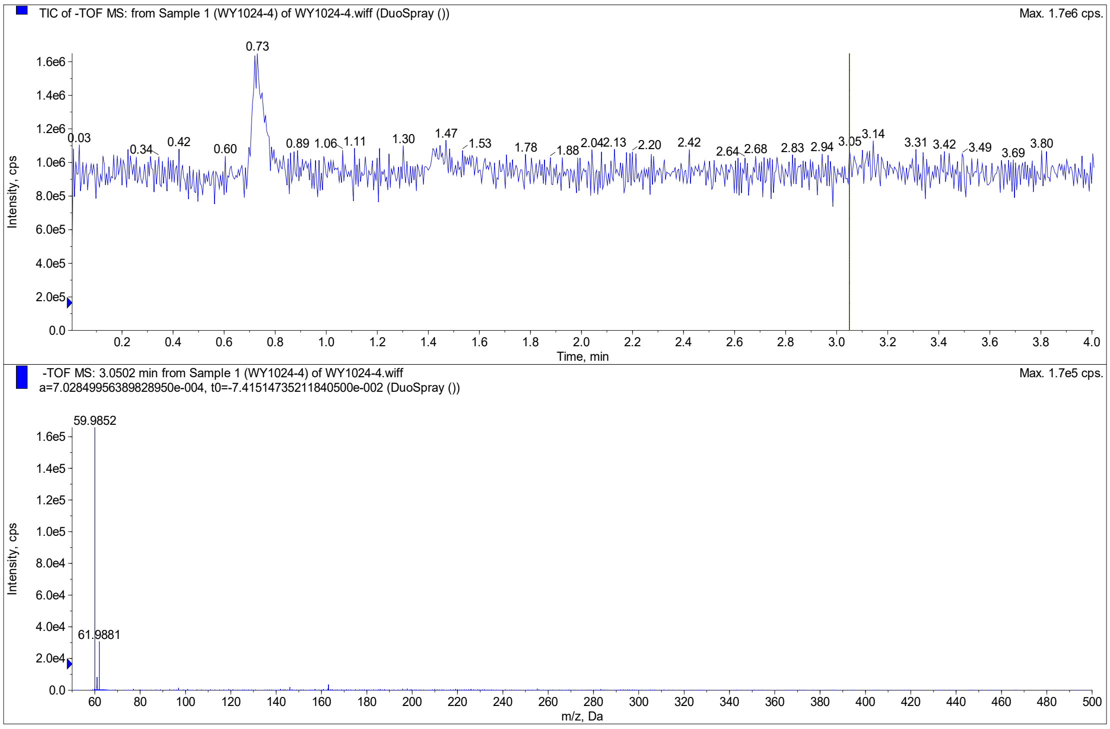

Figure S5. Negative control of cuminic acid in cucumber leaves.

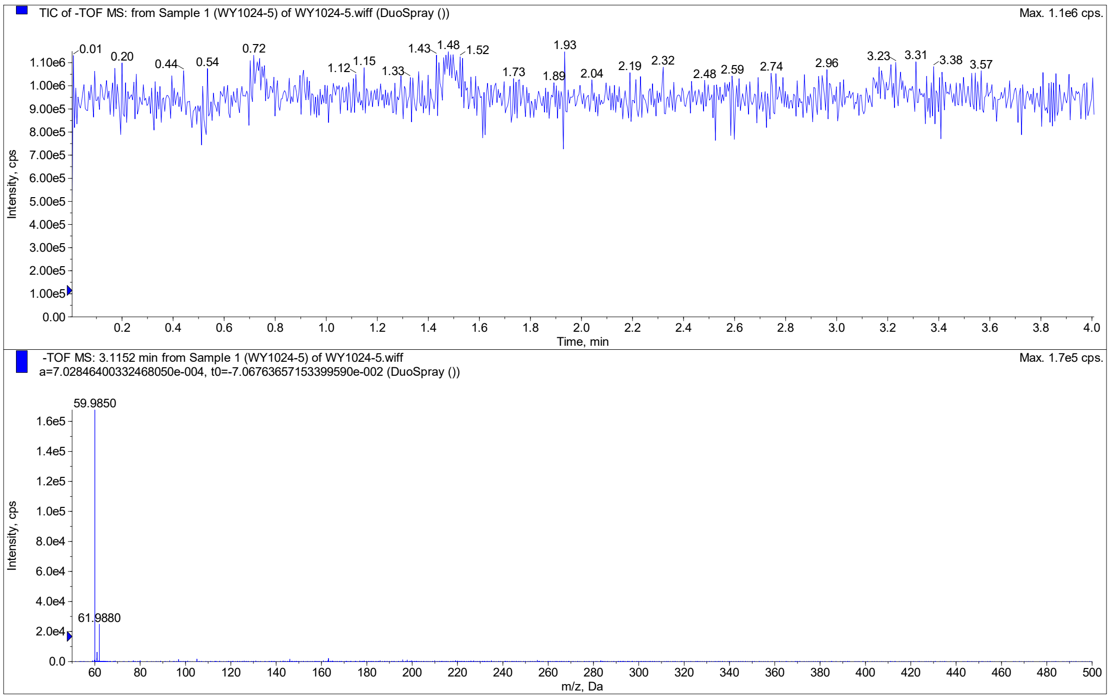

Figure S6. Negative control of cuminic acid in cucumber roots.
